# Quest for Orthologs in the era of Data Deluge and AI: Challenges and Innovations in Orthology Prediction and Data Integration

**DOI:** 10.1007/s00239-025-10272-6

**Published:** 2025-10-14

**Authors:** Sina Majidian, Armin Hadziahmetovic, Felix Langschied, Stefano Pascarelli, Silvia Prieto-Baños, Jorge Rojas-Vargas, Quest for Orthologs Consortium, Sina Majidian, Armin Hadziahmetovic, Felix Langschied, Stefano Pascarelli, Silvia Prieto-Baños, Jorge Rojas-Vargas, Lars Arvestad, Jitender Cheema, Salvatore Cosentino, Ingo Ebersberger, Elena Kuzmin, Yannis Nevers, Nikolai Romashchenko, Maureen Stolzer, Yan Wang, Alex Warwick Vesztrocy, Yuting Xiao, Edward L. Braun, Christophe Dessimoz, Abdoulaye Baniré Diallo, Dannie Durand, Gang Fang, Toni Gabaldón, Natasha Glover, David A. Liberles, Claire McWhite, Erik L. L. Sonnhammer, Aïda Ouangraoua, Irene Julca, Edward L. Braun, Christophe Dessimoz, Abdoulaye Baniré Diallo, Dannie Durand, Gang Fang, Toni Gabaldón, Natasha Glover, David A. Liberles, Claire McWhite, Erik L. L. Sonnhammer, Paul D. Thomas, Aïda Ouangraoua, Irene Julca

**Affiliations:** 1 Department of Computer Science, Johns Hopkins University, 3400 North Charles St., Baltimore, MD 21218 USA; 2 Institute for Informatics, Ludwig-Maximilians-Universität München, 80333 Munich, Germany; 3 Applied Bioinformatics Group, Institute of Cell Biology and Neuroscience, Goethe University, Frankfurt, Germany; 4 Department of Computational Biology, University of Lausanne, 1015 Lausanne, Switzerland; 5 SIB Swiss Institute of Bioinformatics, 1015 Lausanne, Switzerland; 6 Department of Biology, Institute of Molecular Systems Biology, ETH Zurich, Zurich, Switzerland; 7 Department of Biology, University of Western Ontario, 1151 Richmond St, London, ON N6A 3K7 Canada; 8 Department of Biology, University of Florida, Gainesville, FL 32611 USA; 9 Département d’informatique, Université du Québec À Montréal, 201 Av. du Président-Kennedy, Montreal, QC H2X 3Y8 Canada; 10 Department of Biological Sciences, Carnegie Mellon University, 4400 Fifth Avenue Pittsburgh, Pittsburgh, PA 15213 USA; 11 Shanghai Key Laboratory of Cancer System Regulation and Clinical Translation, Jiading District Central Hospital, Renji Hospital Jiading Branch, Shanghai, 201800 China; 12 Life Sciences Department, Barcelona Supercomputing Center (BSC), Plaça Eusebi Güell, Barcelona, Spain; 13 Mechanisms of Disease Department, Institute for Research in Biomedicine (IRB), Barcelona, Spain; 14 Catalan Institution for Research and Advanced Studies (ICREA), Barcelona, Spain; 15 Department of Biology and Center for Computational Genetics and Genomics, Temple University, Philadelphia, PA 19122 USA; 16 Department of Molecular and Cellular Biology, The University of Arizona, Tucson, AZ 85721 USA; 17 Department of Biochemistry and Biophysics, Science for Life Laboratory, Stockholm University, Box 1031, 17121 Solna, Sweden; 18 Department of Population and Public Health Sciences, University of Southern California, Los Angeles, USA; 19 Department of Computer Science, Faculté Des Sciences, Université de Sherbrooke, 2500 Boulevard de L’Université, Sherbrooke, QC J1K 2R1 Canada; 20 Department of Microbiology & Immunology, Schulich School of Medicine & Dentistry, University of Western Ontario, 1151 Richmond St, London, ON N6A 3K7, Canada; 21 CIBER de Enfermedades Infecciosas, Instituto de Salud Carlos III, Madrid, Spain

**Keywords:** Orthology, Gene function, Protein domains, Artificial intelligence, Paralogy

## Abstract

The rapid advancement of DNA sequencing technologies and computational algorithms has led to an unprecedented surge in genomic data, driven by several large-scale sequencing projects worldwide. Orthology plays a crucial role in understanding evolutionary patterns of genes and their functions. At the last Quest for Orthologs meeting (Montréal, Canada—2024), we discussed recent advances in orthology inference, with a focus on the impact of artificial intelligence (AI), protein structures, RNA splicing isoforms, and protein domain evolution together with other evolutionary considerations. A long-standing challenge in the field is the functional annotation of paralogs, for which we present novel approaches. The meeting also emphasised strategies for integrating diverse genetic features into the concept of orthology, encouraging frameworks that account for elements like alternative splicing, domain organisation, and regulatory sequences. We discuss various applications of orthology and paralogy to environmental research, agriculture, and comparative genomics. Additionally, we report recent progress in orthology inference methodologies and resources. This work represents a collaborative synthesis of insights and innovations presented at the 8th Quest for Orthologs meeting, highlighting current progress while outlining future directions for orthology research.

**Supplementary Information:**

The online version contains supplementary material available at 10.1007/s00239-025-10272-6.

## Introduction

As the genome sequencing era facilitated access to the gene repertoire of a growing number of organisms, the goal of annotating the functions of each gene became a pressing need. Classically, one would have performed a set of biochemistry and/or genetics experiments for each gene in each organism to assign functions, but this is clearly intractable at a genome-wide scale. With the broad conservation of genes and proteins across species in sequenced genomes, together with existing biochemical knowledge, it was realised that functional annotations could be transferred between homologous sequences (sequences that descend from the same ancestor) (Koonin 2005). This approach embraces the underlying assumption that functional properties tend to be conserved within gene families.

However, not all homologs are equivalent for the task. In 1970, Walter Fitch (1970) pointed out that homologs can be subdivided into two major subtypes: orthologs, which arise through speciation events, and paralogs, which result from gene duplication. Gray and Fitch (1983) added a third subtype, xenologs, to describe homologs formed through horizontal gene transfer. Darby et al. (2017) further subdivided xenologs, illustrating the potential complexity of evolutionary histories that include transfers. Indeed, the large number of potential histories for homologous proteins presents challenges for any classification (Fitch 2000). Although these categories are defined based on evolutionary history, they do have implications for protein function. Susumo Ohno (1970) suggested that the redundancy created by the gene duplication process enabled faster sequence and functional evolution. While it is recognised that genes that are homologous and have not been duplicated can undergo functional divergence, orthologous genes are more likely to have retained function from an ancestral state (Gabaldón and Koonin 2013). The Quest for Orthologs consortium was born to address the challenges associated with these efforts (Gabaldón et al. 2009).

In July 2009, Erik Sonnhammer and Albert Vilella organised the first 'Quest for Orthologs' meeting at the Wellcome Trust Conference Centre in Hinxton, UK, where they brought together around 30 experts, representatives of the major conceptual developments, methods, and databases in the field of orthology predictions to jointly discuss shared present and future challenges (Gabaldón et al. 2009).

This initial meeting highlighted the vast array of algorithms available for inferring orthologs from genome sequence data, leading to the ongoing development of several algorithms by the community (Gabaldón et al. 2009). Subsequent meetings focussed on establishing standards for reference proteomes, benchmarking datasets (Altenhoff et al. 2024b), and file formats to improve interoperability and reproducibility (Dessimoz et al. 2012; Sonnhammer et al. 2014; Altenhoff et al. 2016; Nevers et al. 2022). As genome sequencing has expanded across the Tree of Life, the QfO community has emphasised scalability, pushing tools and resources to handle the exponential growth in genomic data while accounting for complex evolutionary events such as gene duplications and domain rearrangements (Forslund et al. 2018; Linard et al. 2021; Langschied et al. 2024a). Additionally, applications of orthology were highlighted from gene function prediction, phylostratigraphy, comparative genomics, and phylogenomics (Glover et al. 2019). Recent discussions have also extended orthology concepts beyond the gene level and emphasised democratising access to resources (Linard et al. 2021; Langschied et al. 2024a).

Orthologs can be identified by sequence comparisons followed by graph-based or phylogenetic reconstruction methods (gene tree and species tree reconciliation), which analyse patterns of sequence evolution after divergence (Kristensen et al. 2011; Nevers et al. 2020). Additionally, syntenic information—based on the conservation of chromosomal locations between species—can aid in orthology identification, though it has its limitations. Gene order may be disrupted by events such as genome rearrangements or non-segmental gene duplications, particularly over long evolutionary distances (Langschied et al. 2024a). However, the problem of orthology inference continues to face significant challenges, including scalability limitations when applied to large datasets, computational time constraints, and the persistent difficulty of achieving high-precision orthology predictions.

**Fig. 1:**
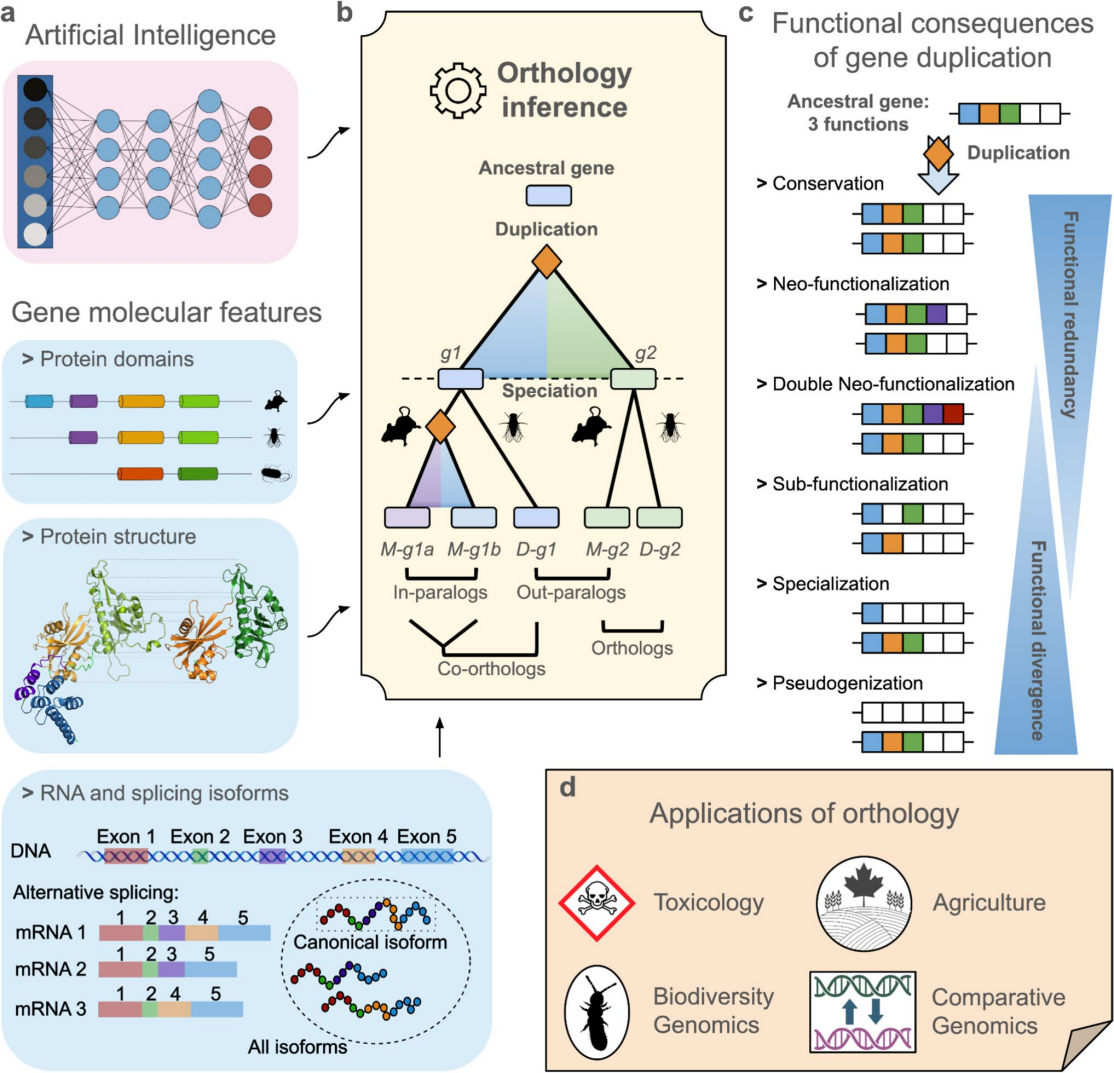
Summary of the QfO8 meeting. **a** Hot topics and future directions in method development and applications within the QfO community, namely artificial intelligence, protein domains, protein structure, RNA and splicing isoforms. **b** Definition of orthology and paralogy, including various paralogous subtypes (e.g. in-paralogs and out-paralogs). **c** Duplications and functional divergence. **d** Applications of orthology

To address these challenges, the 8th Quest for Orthologs (QfO8) meeting was held at the Université du Québec à Montréal on July 17–18, 2024 (see Supplementary Note 1 for the meeting program). In this report, we provide a summary of four invited talks, 20 selected presentations from submitted abstracts, and highlight from the poster sessions. The discussions at the QfO8 meeting mainly focussed on advancing orthology prediction through more scalable algorithms, the future of orthology and artificial intelligence, improving the integration of diverse data sources to the concept of orthology, and extending the practical application of these methods (Fig. 1). Several groups explored the problem of extending the concept of orthology to genetic features that have come to light through advances in sequencing technology, including multidomain proteins, alternate splice variants, and microRNAs. Others presented methodological innovations. Discussions also explored the wide-ranging applications of orthology predictions across different fields, with particular emphasis on their role in functional annotation, comparative genomics, environmental ecology, and biomedical research. The latest updates in tools and databases were presented as essential resources for the scientific community, reinforcing their importance in improving orthology accuracy and accessibility. Looking to the future, the potential of AI-driven approaches and large language models (LLMs) for improved orthology prediction was a topic of spirited debate, although these methods also introduce new challenges in data integration and interpretation. Below, we synthesise the insights from the QfO8 presentations alongside recent literature to provide the current state of knowledge in the study of orthology and its future directions.

## Emerging Problems in Orthology

Orthology was introduced in the context of species tree inference (Fitch 1970), a decade before the Nobel Prize for DNA sequencing. The intervening decades have revealed introns, alternate splicing, catalytic RNAs, and numerous other genomic features that were unknown in 1970. Moreover, the complexity of large gene families necessitated the recognition of one-to-many and many-to-many sets of orthologs. Terminology useful for these cases, such as co-orthologs, in-paralogs, and out-paralogs (Fig. 1b), was introduced by Sonnhammer and Koonin (2002). The continued accumulation of genomic data and advances in analyses have served to emphasise the complexity of orthology relationships. These advances raise the question of how the concept of orthology can be productively extended to accommodate other genetic features. Any new definition should not only reflect the essential properties of the feature in question but also remain consistent with the original notion of orthology. Ideally, such definitions should be formal enough to support algorithm development and unambiguous feature identification.

### Multidomain Orthology

**Fig. 2:**
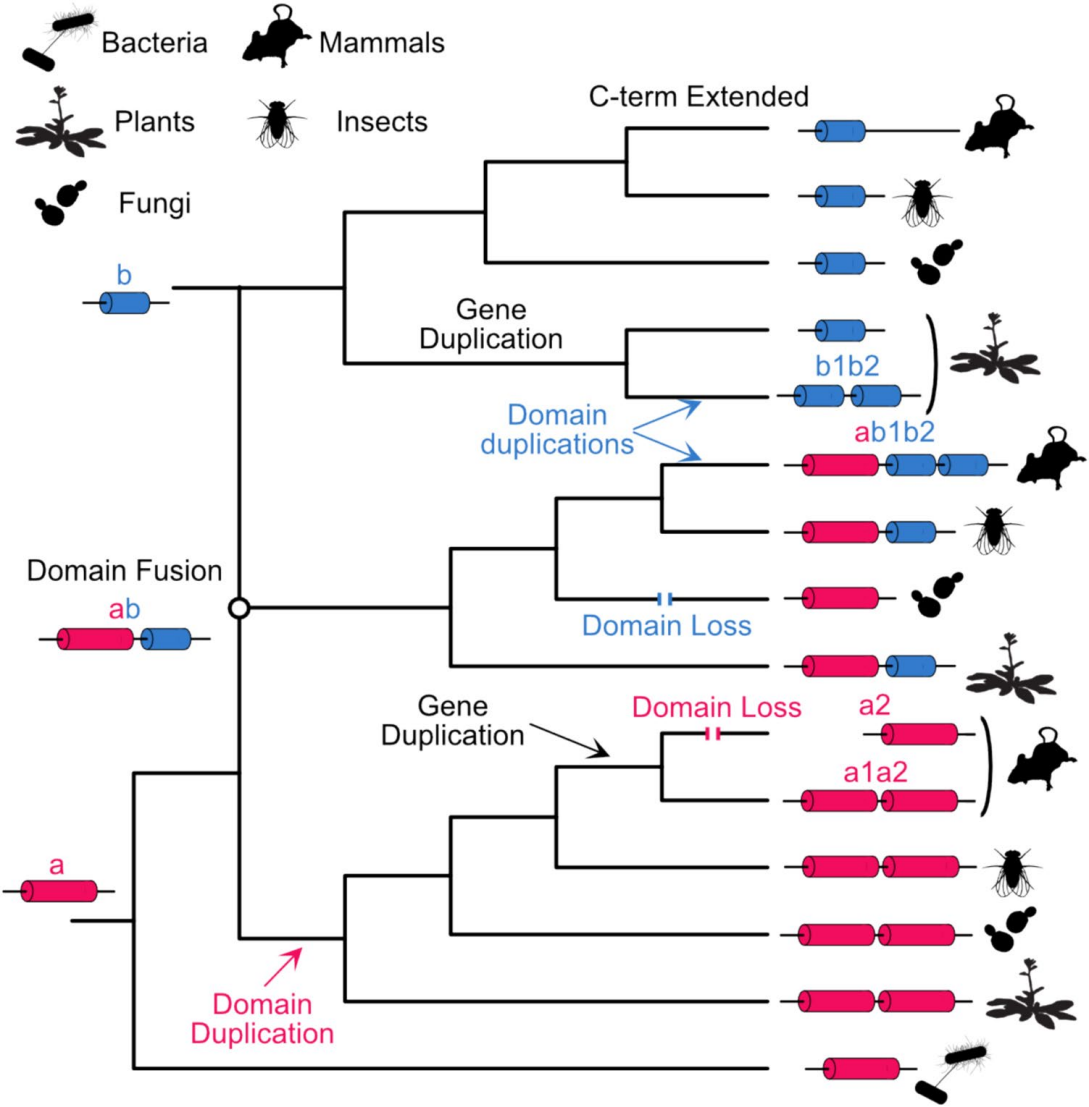
Orthology at the level of protein domains can be very complex. This figure shows the evolution of three hypothetical protein families that include two different domains. The first family corresponds to proteins with one or two copies of domain ‘a’ (red), which is present in bacteria and eukaryotes. The second family comprises proteins with one or two copies of domain ‘b’ (blue), which is limited to eukaryotes. The third family reflects a domain fusion (indicated with a circle) that results in a network-like history at the level of complete proteins. However, the histories of the individual domains remain tree-like (Supplementary Fig. S1). Domain histories can be treelike even when they are parts of multidomain proteins with a network-like history (see Fig. 2). This figure shows hypothetical trees for (a) domain ‘a’ and (b) domain ‘b’ with associated domain structures for the relevant proteinsThe genes encoding proteins with both domains have undergone duplications, creating pairs of in-paralogs in vertebrates (domain ‘a’) and plants (domain ‘b’). Domain duplications have resulted in proteins with ‘a1a2’, ‘b1b2’, and ‘ab1b2’ domain structures. Domain losses resulted in the protein with the ‘a1a2’ structure reverting to a single domain state (‘a2’ in vertebrates) and the protein with the ‘ab’ domain structure reverting to an ‘a’ domain structure (in fungi). The fungal ‘a’ domain protein in the third (‘ab’) family can be distinguished from the ‘a’ domain proteins in the first family because a phylogeny of ‘a’ domains would show that it is nested within the ‘ab’ type protein (as in Braun and Grotewold 2001). In addition, the carboxyl-terminus of the vertebrate ‘b’ domain protein underwent an extension. Additional processes not shown in this figure can also modify domain architecture

Mosaic sequences that encode multidomain proteins are a particularly challenging case for orthology inference. Domains are self-stabilised units formed by secondary structural elements packed together into a hydrophobic core. Proteins typically have conserved functional domains that are important for their activity, and many proteins are characterised by the presence of multiple domains. Domains can be rearranged among and within proteins via duplications, insertions, fusions, and losses (Fig. 2 and Supplementary Fig. S1). As a result, their evolutionary histories are often more complex than those of full-length proteins. Studies of cases where the evidence of specific events is still discernible in the genomic DNA reveal the primary mechanisms mediating changes in domain content: segmental duplication, non-allelic homologous recombination, retrotransposition, non-homologous end joining, exonization of non-coding sequence, transposon-mediated insertion, and read-through errors (Long et al. 2013). As a result, full-length protein comparisons can be misleading when different domains within the same protein have distinct evolutionary trajectories, and orthologs may not have the same domain content (Song et al. 2008; Forslund et al. 2011; Stolzer et al. 2015; Persson et al. 2019; Dohmen et al. 2020). While this is generally rare, it occurs at low frequency even between very closely related species (Forslund et al. 2011). More than two decades ago, Fitch (2000) recognised the challenges associated with defining orthology for proteins with different domain contents, calling it the ‘recombination problem’—a long-standing challenge highlighted by several presentations at QfO8. Some orthology databases, such as the COG (Galperin et al. 2021) or MBGD (Uchiyama et al. 2019), incorporate domain-level concepts for prokaryotes, but there is little work that directly addresses the problem of domain-level orthology in eukaryotes. Three presentations at QfO8 tackled the problem of recognising and interpreting orthology at different levels of protein organisation. Erik Sonnhammer presented InParanoiDB (release 9), the only database explicitly containing domain-level orthologs. This feature enables comparisons between full-length and domain-specific orthologs to investigate evolutionary relationships, revealing cases of discordant domain orthology (Persson and Sonnhammer 2023). To accomplish the goal of inferring orthologous domains that may not be captured in comparisons of full-length proteins, InParanoiDB uses Domainoid (Persson et al. 2019), with domain definitions based on the Pfam database (Mistry et al. 2021). Additionally, InParanoiDB (Persson and Sonnhammer 2022) uses the DIAMOND tool for orthology analysis across the ever-growing number of complete proteomes, significantly reducing runtime compared to traditional tools like BLAST (Altschul et al. 1990).

Lars Arvestad and Dannie Durand focussed on the concept of full-length multidomain orthology. Orthologs that share some, but not all domains, can arise when sequences that share a common ancestor sustain lineage-specific domain gains or losses (e.g. the central family in Fig. 2). Sequences that share some, but not all domains can also arise when instances of the same domain family are inserted into otherwise unrelated sequences in independent events. Both scenarios result in pairs of sequences with at least one domain in common, but only the first scenario corresponds to protein-level orthology (Song et al. 2008). The challenge is to distinguish between the two.

In practical terms, graph structure in a sequence similarity network—where nodes represent protein sequences and edges represent pairwise similarity—can be exploited to distinguish between these cases. Orthologous families appear as dense subgraphs in such networks. Neighbourhood Correlation (Song et al. 2008) distinguishes between dense subgraphs and patterns indicative of domain sharing, outperforming sequence comparison in curated multidomain families (Joseph and Durand 2009). This approach can be further enhanced using synteny information (Ali et al. 2013, 2016). However, scalability is an issue. Lars Arvestad presented recent algorithmic innovations to improve the efficiency of Neighbourhood Correlation. These advances provide a foundation for homology identification in networks of millions of sequences (Durairaj et al. 2023).

Orthology prediction for multidomain families would be greatly advanced by a better understanding of the "design rules" that govern which domain combinations are allowed in functional proteins (Vogel et al. 2005; Cui et al. 2022). Xiaoyue Cui’s preliminary results using natural language embedding techniques separate genuine domain combinations from artificial data representing domain combinations not observed in nature. Her results suggest that embeddings offer a promising model for exploring the roles of domain adjacency and co-occurrence in shaping protein function. Despite these advances, challenges remain in integrating domain-level orthology predictions across large-scale datasets and reconciling conflicting domain evolutionary histories (Stolzer et al. 2015).

Structural information is also important for defining protein domains, and recent advances in protein structure prediction have significantly advanced this field. Christine Orengo presented new work on exploiting computationally predicted structural models (CSMs) for the discovery of previously unknown domains. These results are available in The Encyclopaedia of Domains (TED) resource (Lau et al. 2024), an important source on predicted protein domains which was recently incorporated into the latest CATH release [https://www.cathdb.info] (Waman et al. 2024). However, identifying domain boundaries is challenging, with machine learning showing promising results. For instance, Chainsaw and Merizo, two machine learning methods, outperform other approaches in parsing domains (Lau et al. 2023; Wells et al. 2024). Comparisons of these two tools reveal trade-offs: Merizo tends to "oversplit" proteins, while Chainsaw may "undersplit". Nevertheless, both methods highlight the importance of structural data for protein domain identification, particularly leveraging resources like the AlphaFold Protein Structure Database (Varadi et al. 2022). In summary, the surge in structural data has transformed the protein domain field, opening new possibilities for more precise orthology predictions and improving our understanding of evolutionary relationships at the domain level.

### Transcriptional Orthology in the Context of Alternate Splicing

A comprehensive definition of gene orthology must incorporate information about differences among transcripts of a single gene. Alternative splicing is ubiquitous in some groups of eukaryotes (Pan et al. 2008; Mudge et al. 2011; Reddy et al. 2013), and alternative transcripts can have similar, different, or even opposing functions (Matlin et al. 2005). This complicates orthology inference, as the choice of transcript isoform can influence sequence alignments, gene tree topology, and ultimately ortholog and paralog assignments. Thus, in some cases, considering relationships at the isoform level may provide a more accurate representation of functional equivalence. While transcript isoforms are often conserved across orthologous genes (Baek and Green 2005), substantial variation in expression can exist across tissues within species (Barbosa-Morais et al. 2012; Merkin et al. 2012). One solution to the problem of transcript orthology is the identification of ‘canonical’ isoforms that are both functional and highly expressed (Spitzer et al. 2006). However, identifying these canonical isoforms is complex, and simply selecting the longest isoform can be problematic (Rodriguez et al. 2013; Philippon et al. 2017). Ultimately, gene orthology and paralogy relationships must be assessed from a broader perspective, incorporating more than just canonical isoform sequences.

One obvious way to move beyond canonical isoforms is to treat individual transcripts as terminals in a single phylogeny (Ouedraogo and Ouangraoua 2024a). Yet, the evolutionary history of splicing isoforms can be more complex than gene evolution, as additional evolutionary events can occur at the level of transcripts. Wend Yam D. D. Ouédraogo discussed how to formalise this approach as a gene tree and transcript tree reconciliation problem, where the transcript tree includes speciation, duplication, and transcript creation events (Ouedraogo and Ouangraoua 2024b). To support this framework, TranscriptDB, a dedicated database, is now available with information on inferred orthology and paralogy relationships between splicing isoforms (Ouedraogo and Ouangraoua 2024c). Additionally, a new software for simulating transcript phylogenies is also available, further enhancing the study of transcript evolution (Ouedraogo and Ouangraoua 2024d). These developments raise important biological questions, including estimating transcript creation rates, identifying conserved splice forms, and exploring the convergent evolution of splice forms. A deeper understanding of transcript histories will therefore offer valuable insights for experimentalists studying tissue-specific splice forms. Future efforts will need to incorporate transcript orthology information to improve the accuracy of gene orthology prediction and our understanding of evolution at the transcript level.

### MicroRNA Orthology

Incorporating microRNAs (miRNAs) in the orthology framework is another important challenge. miRNAs regulate gene expression by inhibiting translation or inducing mRNA degradation (Selbach et al. 2008). As key regulators of gene expression, miRNAs show evolutionary conservation, reflecting their critical roles across diverse species. There has been a growing interest in using the presence of shared orthologous miRNAs as rare genomic changes in phylogenetic estimation (Thomson et al. 2014; Langschied et al. 2023). To support this effort, Felix Langschied introduced ncOrtho, a new algorithm for targeted searches for miRNA orthologs (Langschied et al. 2023). These advances are particularly valuable for understanding miRNA and target site co-evolution, highlighting the intertwined evolutionary dynamics of mRNAs and miRNAs (Langschied et al. 2024b) and their importance for orthology prediction. Improved inference of miRNA orthology also has the potential to resolve difficult relationships in the Tree of Life (Langschied et al. 2023).

However, the short length of miRNA sequences makes their identification from genome assemblies particularly challenging. To overcome this, researchers have successfully used text mining approaches for miRNA identification (Kozomara et al. 2019; Schubö et al. 2022). Having collected all published miRNAs, each for a specific species, researchers have been able to fill gaps in missing miRNA annotations across related species. Mapping these data onto phylogenetic trees enables the identification of conserved sequences and their (shared) regulatory roles across species. Despite these advances, most existing miRNA databases remain limited to specific species or taxonomic groups, and many are rarely updated (Kozomara et al. 2019; Guo et al. 2020; Schubö et al. 2022; Clarke et al. 2025). To overcome these limitations, Armin Hadziahmetovic proposed a novel framework, VECTOR, which integrates systematic data extraction from literature, databases, and sequencing data. This comprehensive approach aims to create a robust and scalable resource for miRNA analysis.

## Methodological Innovations in Orthology

The pursuit of the “perfect” orthology inference method remains a central challenge in the field. Since the establishment of the Quest for Orthologs (QfO) meetings, one of the primary objectives has been to drive innovation in computational methods and to discuss the future direction of orthology research. This section highlights presentations about the present and future applications of artificial intelligence in orthology and developments in phylogenetic reconciliation methods that better account for complex gene family histories.

### Artificial intelligence for orthology

Artificial intelligence (AI) has proven to be a powerful tool across many areas of biology, including genome assembly and protein structure prediction (Whalen et al. 2022). More recently, it has been adopted in the area of orthology inference, offering innovative methods to overcome long-standing challenges in the field. For instance, SonicParanoid2 (Cosentino et al. 2024) combines a novel graph-based algorithm with a binary classifier (AdaBoost) to reduce computational time by avoiding unnecessary alignments. It also uses deep learning, specifically Doc2Vec neural network models, to infer orthology at the domain level. The resulting clusters of orthologs are then merged and processed with the Markov Cluster Algorithm (MCL) to generate multi-species orthologous groups (OGs). SonicParanoid2 has been extensively benchmarked against the Quest for Orthologs (QfO) dataset (Altenhoff et al. 2024b), showing both higher speed and accuracy than comparable methods (Cosentino et al. 2024). For more details about the QfO orthology benchmark service, refer to Altenhoff et al. (2024b).

Another example is TOGA (Kirilenko et al. 2023), which integrates gene annotation with orthology inference by leveraging a machine learning framework. It uses a binary classifier based on the XGBoost gradient-boosting library (Chen and Guestrin 2016), trained on human-mouse orthologs from Ensembl Compara. TOGA extracts features such as intronic and intergenic conservation, synteny, and coding sequence similarity to classify gene pairs as orthologs and paralogs (Kirilenko et al. 2023). This enables high-accuracy ortholog identification in closely related genomes, even in cases involving genome rearrangements like translocations or inversions. However, TOGA is primarily designed for pairwise comparisons to a reference genome (e.g. human) and relies on genome alignments, which can be limiting for more distantly related species or large-scale, all-against-all orthology inference. Unlike methods benchmarked with the QfO proteome dataset, TOGA’s accuracy has been evaluated in specific scenarios using whole genomes. Nonetheless, it demonstrates the power of AI-driven orthology inference.

**Fig. 3:**
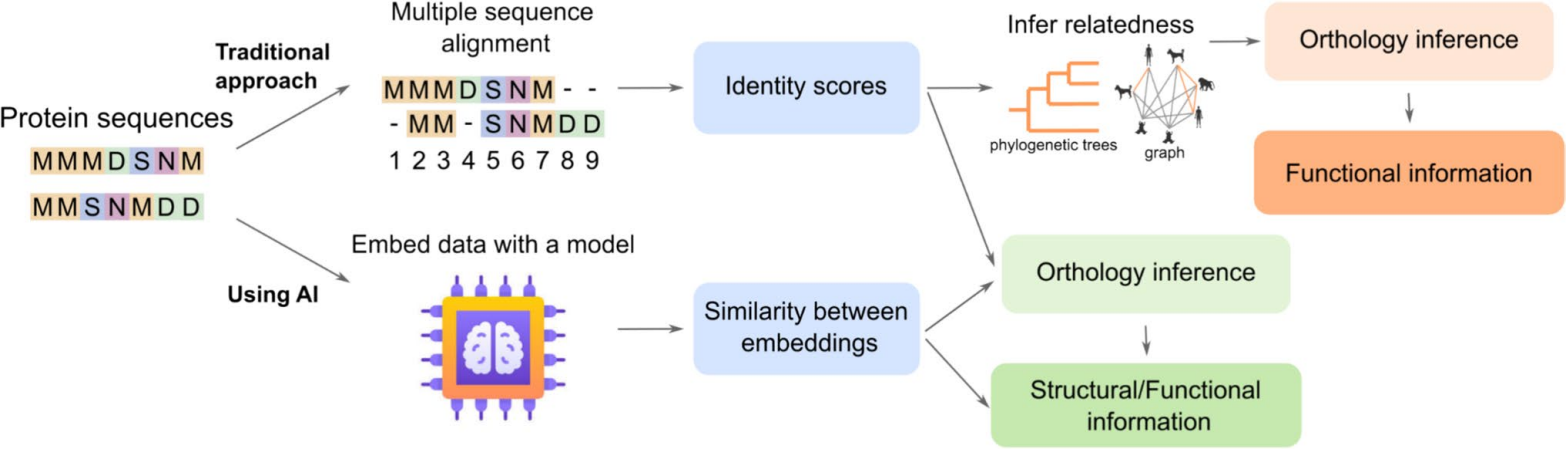
Artificial intelligence (AI) for orthology. Comparison of traditional and AI-based methods for inferring orthology from protein sequences. The traditional approach relies on MSA and identity scores to infer relatedness via phylogenetic trees or graphs, which then support orthology inference and functional annotation. In contrast, AI-based approaches can use deep learning models, such as protein language models, to generate orthology inferences through similarity in embedding space, with direct implications for structural and functional predictions. This new paradigm complements or bypasses alignment-based methods and highlights the growing role of AI in orthology prediction

Protein Language Models (PLMs), a specialised type of large language model, represent a major breakthrough (Nijkamp et al. 2023). PLMs treat protein sequences analogously to human language, interpreting mutations as semantic variations and capturing rich contextual information about functions and structure (McWhite et al. 2023). Building on these recent advances, Claire McWhite showcased how PLMs can improve sequence alignment methods, an essential step in orthology prediction (Fig. 3). Leveraging transformer architectures to represent amino acid sequences as high-dimensional vectors yields embeddings that encode both identity and contextual information, forming a compact, scalable, and biologically informative representation of proteins. This approach allows for fast similarity searches without the need for computationally expensive all-vs-all comparisons and offers a compressible way to store large protein datasets using tools like Facebook’s Faiss library (Douze et al. 2024). Importantly, these embeddings can capture aspects of structure and function even when sequence similarity is low, highlighting the need to decouple functional inference from raw sequence identity. The method also supports mutation analysis within this linguistic framework, where functionally neutral mutations correspond to semantically equivalent "sentences". For multiple sequence alignment (MSA), McWhite et al. (2023) employed embedding-based vector clustering, requiring graph manipulation (e.g. cycle removal and topological sorting) to reconstruct column order. This resulted in improved alignment quality, outperforming tools like MUSCLE and CLUSTAL Omega by 5–10% when sequence similarity is low (McWhite et al. 2023).

AI is also opening new possibilities for domain-based orthology inference. Techniques originally developed for natural language processing, such as Word2vec (Mikolov et al. 2013), are being repurposed to model domain architectures (see Sect. 1.1). These approaches encode proteins as high-dimensional vectors that reflect the presence and arrangement of their domains, while respecting biological constraints on domain number and combinations (McWhite et al. 2023 . By embedding domain structures in this way, researchers can effectively identify patterns in domain combinations and potentially refine orthology predictions in proteins where sequence alone falls short.

AI is also reshaping network-based orthology inference. Graph embedding techniques such as Node2vec and DeepWalk leverage network proximity to infer protein functions (Grover and Leskovec 2016; Jeyaraj et al. 2024). These embeddings, when combined with sequence-based embeddings, have shown improved accuracy in tasks like subcellular localisation and protein function prediction compared to sequence-based methods alone (Szklarczyk et al. 2025).

Together, these developments highlight the transformative potential of AI in orthology. By moving beyond traditional sequence similarity and incorporating context-aware representations through PLMs and other embeddings, AI-driven approaches promise to enable more accurate, scalable, and biologically meaningful predictions. These methods not only enhance core steps such as alignment and domain architecture analysis but also open the door to new ways of thinking about protein evolution and function. As the field continues to adopt and adapt these tools, AI is expected to play a pivotal role in the development of the next generation of orthology prediction methods.

### Phylogenetic Reconciliation

Topological incongruence between gene and species trees is evidence of a range of evolutionary events, including gene duplication, incomplete lineage sorting (ILS), introgression, hybridisation, or horizontal gene transfer. Phylogenetic reconciliation—fitting a gene tree to a species tree—exploits this evidence to infer duplications and transfers, thus distinguishing orthologs from paralogs and xenologs (Goodman et al. 1979). Building on this fundamental approach, incorporating additional evidence, such as gene order, has become a fertile area of research. For instance, Mattéo Delabre introduced a novel method for syntenic reconciliation, called Synesth, to infer evolutionary relationships between sets of co-localised genes across species trees (Delabre and El-Mabrouk 2024). This method reconstructs gene-block histories while accounting for evolutionary events such as duplications, horizontal gene transfers, and losses.

Another promising direction involves extending phylogenetic reconciliation to a broader set of evolutionary processes. While gene tree incongruence is indicative of gene duplication, ancient population processes, including ILS and introgression, can also result in gene trees that disagree with the species tree (Page 1994). If these processes are not accounted for, reconciliation will infer duplications where none occurred, leading to incorrect interpretation of orthologs as paralogs (Stolzer et al. 2012). To address this, a newly developed algorithm reconciles gene trees while accounting for both gene duplication and ILS. This extracts ILS-related gene tree statistics across entire clades in a computationally efficient manner, allowing for species tree-wide characterisation of assortment in ancestral populations.

Together, these advances underscore the importance of reconciliation methods in capturing the full spectrum of evolutionary processes, thereby enabling more accurate orthology assignments.

## Theme 3: Duplications and Paralogous Genes

The quest for orthologs, driven by the idea that identifying orthologs aids in the functional annotation of proteins, has a flip side: the study of non-orthologous proteins, including de novo proteins, xenologs, and paralogs. Specifically, this section focuses on the origin and retention of paralogs, as well as the relationship between gene duplication and protein function.

### Origin and Retention of Duplicated Genes

Gene duplication is a key driver of evolution (Ohno 1970; Wolfe 2001). It occurs through various mechanisms, including unequal crossing over, retrotransposition, duplicated DNA transposition, and whole-genome duplication (WGD) (Magadum et al. 2013). The evolutionary fate of duplicated genes has been extensively studied and widely debated (Prince and Pickett 2002; Birchler and Yang 2022). In this context, Manuel Lafond introduced a digital simulation framework that models functional outcomes based on gene expression and inhibition levels. This approach identified six key evolutionary paths for paralogs: pseudogenization, neofunctionalization, double-neofunctionalization, conservation, subfunctionalization, and specialisation (Fig. 1c) (Kalhor et al. 2024). Interestingly, using the Aevol platform to simulate genome evolution, this study revealed that conservation, neofunctionalization, and pseudogenization are the most probable fates, while subfunctionalization, specialisation, and double-neofunctionalization are less frequent, bridging theoretical predictions with observed patterns (Knibbe 2006; Kalhor et al. 2024). All of these fates evolve with time-dependent rates determined by the mutational opportunity for changes to accumulate. Functional changes in duplicates are also influenced by factors like stoichiometric balance, which can either constrain or promote subfunctionalization depending on duplication type and population size. These dynamics, particularly pronounced in whole-genome duplications, enhance paralog retention and drive functional divergence over time (Rastogi and Liberles 2005; Wilson and Liberles 2023a).

Gene duplicates evolve rapidly at both sequence and functional levels, with their retention shaped by the properties of their functions. The "duplicability" of genes—the likelihood of their retention following duplication—depends on factors such as modularity enabling subfunctionalization, dosage selection at functional and stoichiometric levels, and the potential for functional innovation. These principles form the foundation of the gene duplicability hypothesis (Li et al. 2016), which has been applied to species like Atlantic salmon, which underwent two rounds of whole-genome duplication (Lien et al. 2016; Wilson and Liberles 2023b). A more specific example was the study of the evolution of the myostatin signalling pathway in mice, zebrafish, and Atlantic salmon, where whole-genome duplication produced multiple gene copies that evolved through divergence or interaction (Lawson et al. 2012). David Liberles presented preliminary structural work that shows that the myostatin duplicates in salmon have started to diverge in their interaction specificity with partner proteins, leading to functional diversification. Integrating phylogenetic and protein structural analyses allow tracing the patterns of gene loss and functional divergence, demonstrating how duplication facilitates the evolution of independent pathways while reducing cross-talk among homologs. These studies emphasise the time-dependent dynamics of gene duplication and retention (Konrad et al. 2011), revealing its impact on driving functional innovation and shaping evolutionary trajectories.

### Functional Annotation of Paralogs

After a gene duplication event, multiple co-orthologs may exist—paralogous genes in one species that are orthologous to a single gene in another species (Lechner et al. 2011). Moreover, paralogous genes often exhibit divergent functions, complicating functional annotation. Addressing this challenge, large-scale phylogenetic frameworks have advanced the systematic annotation of protein function by incorporating evolutionary models for orthologs and paralogs. Paul Thomas presented an approach that anchors functional predictions in evolutionary history rather than simple pairwise comparisons. Utilising reconciled gene trees from the PANTHER database (Thomas et al. 2022), expert curators construct explicit models of evolutionary changes by modelling gains and losses of functional characteristics along branches of a gene tree. This approach has now been applied to all ~ 6500 families that include human protein-coding genes, to create a comprehensive resource for human gene functions (Feuermann et al. 2025). The evolutionary models can be applied more broadly to entire databases of sequences by placing them in PANTHER gene trees, using software like TreeGrafter (Tang et al. 2019). TreeGrafter has been integrated into the InterProScan software package (Blum et al. 2025), and the resulting annotations are now provided by major biological databases including UniProt [uniprot.org/database/DB-0052] and NCBI RefSeq [ncbi.nlm.nih.gov/refseq/annotation_euk/process/]. This annotation system has achieved ~ 80% functional annotation coverage for protein-coding genes across vertebrate genomes, and substantial coverage of other eukaryotes.

The reliability of these functional annotations is further enhanced by integrating the concept of the Least Diverged Ortholog (LDO) conjecture, which posits that after gene duplication, the copy with the shorter branch length (LDO) is more likely to retain the ancestral function than the more diverged copy (MDO). This concept is used for defining subfamilies in the PANTHER database. However, small differences in branch lengths may not reliably distinguish between LDOs and MDOs. To address this, Alex Warwick Vesztrocy presented a statistical method for detecting significant rate shifts in branch lengths following gene duplication events. By integrating expression data as a functional feature, this study showed that LDOs tend to preserve ancestral functions, while MDOs often diverge functionally. This approach proposed LDOs as optimal candidates for cross-species functional annotation transfer, offering great potential for improving the accuracy and reliability of protein function prediction methodologies (Warwick Vesztrocy et al. 2025).

Protein-level analyses have further elucidated the mechanistic basis of paralog evolution by examining protein abundance dynamics and subcellular localisation patterns. High-content single-cell microscopy screens in the budding yeast, coupled with deep learning neural network analysis, revealed proteins that redistribute their protein abundance and subcellular localisation in response to their paralog deletion, thereby identifying asymmetric responses in paralogous protein pairs (Dandage et al. 2023; Greco et al. 2025). These findings indicate that individual paralogs can undergo targeted redistribution or establish novel interaction networks across subcellular compartments.

Model organisms like *Saccharomyces cerevisiae* are key to studying post-duplication protein evolution, thanks to their rich functional genomics datasets. Leveraging this resource, Elena Kuzmin used Synthetic Genetic Array (SGA) methodology—a high-throughput technique that automates the generation and analysis of yeast double mutants—to systematically map genetic interaction networks in post-WGD paralogs through genome-wide screens. This work revealed complex functional relationships and genetic redundancy patterns (Costanzo et al. 2019; Kuzmin et al. 2020). The findings show that WGD paralogs can exhibit a range of evolutionary outcomes, including redundancy, divergence, specialisation, compensation, or dependency, with about one-third displaying functional overlap and compensatory interactions (Kuzmin et al. 2020). Evolution at the gene expression level typically shows redundancy decreasing over time, with observations depending on the time since the duplication event (Hermansen et al. 2016).

Inspired by protein fitness studies and the relationship between selection and function, Gang Fang presented a framework using a two-dimensional plot, with protein length on the x-axis and sequence similarity on the y-axis, to evaluate discontinuous protein evolutionary patterns. This facilitates unsupervised spectral clustering to distinguish “signal” proteins—likely functionally consistent orthologs, including in-paralogs—from “noise” (distant homologs or unrelated sequences) (Yang et al. 2025). This approach accommodates diverse selective pressures and evolutionary histories, bypassing the need for predefined thresholds like e-values or sequence identity in selecting candidates for phylogenetic reconstruction or clustering. Central to this method is the Signal Jaccard Index (SJI), which quantifies functional similarity by measuring signal overlap between protein pairs. SJI constructs a weighted protein network, clustering orthologous groups while incorporating in-paralogs and distinguishing core, high-confidence orthologs from peripheral groups with inconsistent predictions (Yang et al. 2025). This SJI, a two-step adaptation of the BBH (Best Bidirectional Hit) approach, enhances orthologous group identification, providing a more precise and scalable framework for protein function analysis (Yang et al. 2025).

In brief, biological function is a highly multifaceted concept for which a broad consensus definition remains elusive. At its heart lie biochemical parameters associated with concentration (expression level and location) and molecular interactions. Model organism studies, with high-quality experimental annotations, have proven especially valuable in elucidating paralog functional dynamics. Given the enriched genetic and phylogenetic evidence supporting identical or similar biological functions among some paralogs (or in-paralogs), the clustering of functionally consistent in-paralogs can serve as an effective benchmark for orthologous group functional conservation. The integrated approaches, including computational simulations, phylogenetic analyses, and 2D protein evolution mapping, provide a quantitative framework for investigating paralog functional evolution (Yang et al. 2025). Taken together, these methodological advances mark a significant step towards achieving a more nuanced and comprehensive understanding of biological function conservation and divergence following gene duplication.

## Theme 4: Orthology Applications

The applications of orthology and paralogy are central to the goals of the orthology community, as they directly inform method development and ensure a feedback loop in which practical use cases reveal previously unrecognised challenges, which in turn lead to computational improvements. Collaborative interactions between users and method developers were a major focus of QfO8 and emphasised the importance of connecting different research communities.

While the benefits of orthology analysis are frequently framed in a biomedical context, there is a growing focus on the relevance of orthology to agricultural, environmental, and ecological applications (Langschied et al 2024a, b). Markus Hecker presented work on omics-driven toxicology, in which he showed how transcriptomics and proteomics enable better assessment of chemical risks by identifying molecular targets and toxicity pathways (Alcaraz et al. 2025). For example, ethinylestradiol-induced oestrogen receptor disruption in fish illustrates how tools such as SeqAPASS (Doering et al. 2018) can predict species susceptibility to chemicals in different taxa based on protein similarity. However, incomplete functional annotation remains a key challenge in non-model species, where genome duplications and sparse sequencing hinder differential gene expression analysis. Expanding taxonomic coverage and improving functional annotation are necessary steps for better ecological risk assessment.

Another example of environmental applications came from exploring the role of soil invertebrates in the carbon cycle using biodiversity genomics. Ingo Ebersberger presented a use of the target ortholog search tool fDOG and UMAP visualisation (Tran et al. 2025) to simplify the phylogenetic profiles of over 18,000 taxa, where he and colleagues were able to identify clusters of species with different patterns of presence and absence of plant cell wall-degrading enzymes, revealing functional differences among taxa (Tran et al. 2025). Notably, the soil invertebrate *Folsomia candida* (a springtail) was identified as capable of degrading plant cell walls, suggesting a direct role in carbon cycling; currently, animals are often overlooked as ecologically relevant in models. Additionally, the Hydrocarbon Aerobic Degradation Enzymes and Genes (HADEG) database (Rojas-Vargas et al. 2023) was introduced as a curated collection of hydrocarbon degradation genes and enzymes, offering experimentally validated data for petroleum degradation, plastic degradation, and biosurfactant production in bacteria and fungi.

Orthology applications also extend to agriculture, where comparative approaches can be invaluable for understanding key traits (Julca et al. 2021). Based on the QTLSearch algorithm (Warwick Vesztrocy et al. 2018), a phylogeny-aware framework for analysing Quantitative Trait Loci (QTL) data, called ortho-QTLSearch, was developed to identify candidate genes for agriculturally relevant traits such as yield, fruit size, and disease resistance. The method narrows down candidate genes in species with limited functional annotations and promises to improve crop breeding programmes.

Orthology plays a critical role in uncovering the evolutionary histories of genes and genomes across diverse taxa. Conserved gene orders and co-localised genes can enable ancestral genome reconstructions and shed light on chromosome evolution. Charles Bernard presented EdgeHOG, a method for inferring ancestral gene orders (Bernard et al. 2025), which uses hierarchical orthologous groups (HOGs) (Sarton-Lohéac et al. 2025) to trace gene adjacencies back through evolutionary trees. Its linear-scale efficiency enabled the reconstruction of 1,133 ancestral genomes across the Tree of Life and provided insights into genomic rearrangements and the evolution of co-localised genes (Bernard et al. 2025).

Collectively, these studies demonstrated the wide-ranging applications of orthology, from environmental research and bioremediation to agriculture and evolutionary biology. Recent advances in orthology methods and tools facilitate large-scale genomic analyses; however, key challenges such as broader taxonomic coverage and better functional annotation remain. These developments underscore the importance of orthology in solving real-world biological challenges and advancing our understanding of biodiversity and genome evolution.

## Orthology Tools and Challenges

### Orthology Tools and Database-Update

At the core of the QfO community, several long-standing orthology resources are being updated. InParanoidDB 9 has recently undergone major updates (Persson and Sonnhammer 2023), which now include over 1 billion orthologous groups spanning 640 species and introduce the domain orthologs (see Sect. 1). Both full-length and domain orthologs were inferred using the InParanoid-DIAMOND algorithm (Persson et al. 2019; Persson and Sonnhammer 2022), which is over 700 times faster than InParanoid-BLAST, while maintaining comparable benchmark quality. InParanoiDB is the only ortholog database offering explicit global domain-level orthology across the Tree of Life, and a completely new website has been developed to facilitate the search and visualisation of domain-level orthologous groups. Additionally, this database’s predicted pairwise ortholog relationship has been used to expand knowledge of the protein interactome from a few model species to all species available in FunCoup (Buzzao et al. 2024). Damian Szklarczyk introduced the new STRING database v12.0, featuring an overhauled protein function transfer system by propagation (Szklarczyk et al. 2023) and enabling users to upload proteomes for automated orthology inference and functional annotation. In addition, STRING now offers pre-computed embeddings of sequence and protein network data, facilitating downstream machine learning applications (Szklarczyk et al. 2025).

The community has taken an important step towards expanding database sizes to match the rapid pace of genome sequencing. The two SwissOrthology resources, OrthoDB and OMA, have undergone important changes. Evgeny Zdobnov reported significant increases in the coverage and diversity of species in the OrthoDB databases. OrthoDB version 12 now includes pre-computed orthology for 5,827 eukaryotic and 18,158 prokaryotic genomes (Tegenfeldt et al. 2024), while OMA has expanded to 2,927 genomes (Altenhoff et al. 2024a). OrthoDB serves as the basis for BUSCO (Tegenfeldt et al. 2024), a widely used tool for assessing genome assemblies, which is now faster with miniprot at its core (Li 2023). Yannis Nevers introduced OMArk, a novel tool for evaluating genome annotation completeness (Nevers et al. 2024). Ikuo Uchiyama reported that the Microbial Genome Database (MBGD) now includes 34,097 genomes—more than double the previous release from 2022 (Uchiyama 2003). OrtholugeDB, which leverages phylogenetic distance ratios to infer and evaluate orthologous pairs, covers over 2,000 species (Whiteside et al. 2013). Finally, PhylomeDB, a well-curated repository of annotated sequences, alignments, and gene phylogenies (phylomes), has grown to include more than 8 million gene trees and 6,000 species (Fuentes et al. 2022).

### New Orthology Resources

Aside from tool and database updates, several new resources were presented at the meeting. Fiona Brinkman introduced IslandCompare, a tool for detecting and visualising Genomic Islands, clusters of genes likely originating from horizontal gene transfer, often enriched with virulence factors or antimicrobial resistance genes (Bertelli et al. 2022). For microbial genomes, Evgeny Zdobnov presented LEMMI, a standardised platform for benchmarking metagenome composition assessments (Seppey et al. 2020). Lastly, Stefano Pascarelli described FastOMA, a scalable orthology inference tool capable of analysing over 2,000 UniProt eukaryotic reference species in less than 24 h using 300 CPUs (Majidian et al. 2025). FastOMA employs fast k-mer-based mapping of input proteins onto OMA hierarchical orthologous groups (HOGs; reference gene families) and uses taxonomy-guided gene tree reconstruction to distinguish orthologs from paralogs efficiently. The resulting orthology information is provided in OrthoXML, a widely accepted and richly structured format for evolutionary analysis (Schmitt et al. 2011; Yazdizadeh Kharrazi et al. 2025).

Cristine Orengo discussed how advances in protein structure prediction can be leveraged to enhance our understanding of orthology. Protein structure has long been recognised as a valuable source of evolutionary information. It offers a deeper level of conservation than protein and gene sequences, which has enabled the identification of distant homologs in the “twilight zone” of divergence (Rost 1997) and defined protein domain boundaries (Orengo et al. 1997). However, the use of experimentally determined structures in evolutionary analyses has been limited by their relatively slow acquisition; as of 2024, there are just over 227,000 experimentally resolved structures (Burley et al. 2024). While impressive, this number is now dwarfed by the over one million computationally predicted structural models (CSMs) available (Burley et al. 2024). This explosion of CSMs is the result of groundbreaking advances in the field of computational protein structure prediction, driven by tools like AlphaFold (Jumper et al. 2021), RoseTTAFold (Baek et al. 2021), and OpenFold (Ahdritz et al. 2024).

In summary, the orthology databases and tools that are being developed underscore the community's dedication to advancing orthology research. Orthology databases now span thousands of genomes, increasing species diversity, while new tools address critical challenges in the field. Together, these resources provide researchers with robust platforms to tackle the growing complexity of genomic data and advance the study of orthology and gene function.

### Annotation Matters

Recent advances in sequencing technologies, orthology methods, and downstream applications have greatly expanded our understanding of genomes. However, the quality of gene repertoires often lags behind, posing significant challenges. With a few exceptions, such as TOGA (Kirilenko et al. 2023), protein-coding gene repertoires are the foundation material needed to infer orthology. However, accurately identifying all protein-coding genes within a genome—part of the process known as structural gene annotation—remains a bottleneck for orthology inference. Thus, inaccuracies in gene annotation can lead to complications and false downstream results, including the spurious classification of evolutionary events (Bányai and Patthy 2016; Weisman et al. 2020).

The gene annotation method significantly impacts orthology inference, affecting the quality of both pairwise orthologs and hierarchical orthologous groups. Silvia Prieto-Baños presented empirical evidence that highlights the extent of this problem and showed that differences are especially pronounced when comparing gene repertoires derived from purely ab initio annotation methods with those generated by comprehensive pipelines that integrate ab initio predictions, homology-based approaches, and transcriptomic evidence. Furthermore, discrepancies were observed even among well-established annotation resources such as RefSeq, Ensembl, and UniProt (Prieto-Baños et al. 2025). Despite the critical impact of annotation sources, this issue has received limited attention in comparative genomics and other applied studies, leaving a significant gap in understanding and addressing its implications. Moreover, the quality of orthology inference results could be used as a way to evaluate annotation quality when several assemblies are annotated in parallel.

Early quality assessment is crucial for ensuring the accuracy of downstream analyses. While it is common practice to perform a quality control step early in the workflow, it is often limited to filtering out low-quality data. However, noise remaining after this filtering step may still introduce errors. For example, assembly gaps can mimic gene loss, and contaminations can give a false impression of gene gain. It is therefore essential to make quality control metrics accessible throughout the analysis. To support this, Felix Langschied introduced the web-based genome portal: G-nom [https://ebersberger-46-155.biologie.uni-frankfurt.de/gnom/]. G-nom allows users to collect quality metrics on species-specific pages, which can be organised in dashboards to provide a comprehensive overview of data quality across custom taxon sets. It integrates genome assembly statistics such as the N50 value and contig length distributions with gene set completeness metrics like BUSCO (Tegenfeldt et al. 2024), OMArk (Nevers et al. 2024), and fCAT (Tran and Ebersberger 2022). Additionally, G-nom is fully integrated with taXaminer, a software for the interactive exploration of taxonomic diversity in genome assemblies [https://github.com/BIONF/taXaminer]. The 3D visualisation produced by taXaminer enables the identification of genes introduced through contamination and the distinction of those from genes gained via horizontal gene transfer.

Many quality assessment methods rely on core gene comparisons (e.g. OMArk, BUSCO), and therefore, they can be affected by biases caused by an unbalanced taxon sample when determining core genes. Evaluating gene repertoires using assembly-intrinsic information and orthology inference results provides a novel, reference-agnostic approach to quality assessment.

### Dealing with the Data Deluge

Rapid advances in science have generated an unprecedented explosion of genomic data and the complexity of evolutionary relationships. However, valuable historical data risks being lost, and its recovery and preservation efforts continue to face significant challenges. For instance, while artificial intelligence, including Large Language Models (LLMs), offers promising tools for extracting information, scanning old documents and handling special characters remain major obstacles. Additionally, incomplete metadata, such as missing information on taxon, time, tissue, or geographical origin, can limit data usability (Peterson et al. 2018), and inconsistencies in gene naming, with each author adopting unique conventions, further complicate data retrieval and analysis. These issues highlight the ongoing challenge of effective data management in the scientific community following central principles of FAIR (Findable, Accessible, Interoperable, Reusable) data (Wilkinson et al. 2016; Dessimoz and Thomas 2024). For instance, automated preprocessing steps, data filtering, and reduction can help in removing low-quality data and reducing the data size while maintaining essential information. However, this data reduction usually comes at the cost of information loss. An example is the NCBI’s initial proposal to remove base quality scores from the Short Read Archive (https://grants.nih.gov/grants/guide/notice-files/NOT-OD-20-108.html, accessed 22 July 2025), despite their known utility for variant calling, featuring this trade-off (Ochoa et al. 2017). In parallel, the long-term accessibility of bioinformatics resources is threatened by URL decay, with approximately 27% of resource links becoming non-functional over time, regardless of their content type (Wren et al. 2017). A more recent analysis of web services, including some related to orthology, found that 25.7% of the tools were no longer reachable (Kern et al. 2020). Together, these examples emphasise that maintaining long-term data integrity and accessibility requires not only technological innovation but also efforts in infrastructure development, sustainable funding, and curation practices.

Another pressing challenge is analysing and extracting meaningful insights from the overwhelming data deluge. For instance, homology searches alone are insufficient to reliably assign gene names and functions. High-quality orthology predictions can address this gap, but the accuracy of these predictions directly impacts the results. To improve outcomes, Fiona Brinkman emphasises the importance of clearly defining the specific problem or analysis, as this allows for the selection of the most appropriate data features, such as protein sequences or structures, for accurate orthology prediction. Once the problem is defined, strategic subsampling and visualisation tools play a pivotal role in interpreting complex biological data. Moreover, greater education is needed on the risks of falsely predicted orthologs and on enhancing the ability to derive meaningful insights from large datasets.

In addition to data recovery and interpretation challenges, orthology inference also faces several technical and methodological limitations. A major concern is the scalability of current methods, as many algorithms struggle to process the ever-growing number of genomes efficiently without compromising accuracy (Cosentino et al. 2024). As datasets expand in size and complexity, the computational cost of orthology prediction increases significantly, demanding more optimised and parallelised approaches. Adding to this, there remains a lack of consensus between orthology inference methods, often leading to conflicting results across databases and tools, which hinders reproducibility and user confidence (Altenhoff et al. 2020). Furthermore, integrating diverse data types, such as proteins, transcripts, protein domains, and structural information, poses technical challenges in standardisation, interoperability, and interpretation (SIB Swiss Institute of Bioinformatics RDF Group Members 2024). The efforts to deal with these challenges are further constrained by data storage and transfer limitations, especially when handling large-scale sequence alignments, gene trees, or structural models. Addressing these obstacles will be critical to improving the potential of orthology-based analyses in genomic research.

## Conclusion and Future Perspectives

As orthology remains central to comparative genomics and functional annotation, ensuring accurate and reliable predictions continues to be a critical step. Expanding the concept of orthology to incorporate additional genomic features—such as multidomain proteins, alternative splice variants, and microRNAs—introduces new challenges, many of which were explored during the meeting. Another key topic was the unprecedented availability of structural data and its implications for orthology inference. Advances in protein structure prediction have improved domain detection and enabled new strategies for identifying orthologs, particularly in the “twilight zone” of sequence identity, where structural and functional conservation persist despite low sequence similarity. Gene duplication adds another layer of difficulty to functional prediction, and its evolutionary consequences were the subject of extensive discussion.

Applying artificial intelligence to orthology prediction is one of the most exciting prospects for future work. Work presented at QfO8 illustrates the power of AI for protein structure prediction, precise domain identification, and sequence alignment, prerequisites for many current orthology pipelines. Domain architecture embeddings open new avenues for improving orthology predictions in multidomain proteins. At the other end of the pipeline, interoperability with machine learning resources to facilitate downstream analyses was also discussed (Szklarczyk et al. 2025). Recently, AI methods have been applied to integrate orthology analysis with gene annotation (Kirilenko et al. 2023). Some of the first work to apply artificial intelligence to orthology inference directly is just coming on line (Cosentino et al. 2024), heralding future innovation to overcome long-standing challenges in the field.

While new tools and updated databases continue to expand the capabilities of orthology inference, their impact depends on broader awareness and stronger communication across the scientific community. Promoting shared standards and highlighting the importance of orthology will ensure that orthology remains a powerful and reliable framework for understanding gene function and evolution.

## Supplementary Information

Below is the link to the electronic supplementary material.Supplementary Fig. S1. Domain histories can be treelike even when they are parts of multidomain proteins with a network-like history (see Fig. 2). This figure shows hypothetical trees for (a) domain ‘a’ and (b) domain ‘b’ with associated domain structures for the relevant proteins.

Supplementary file1 (PDF 47 KB)

Supplementary file2 (DOCX 85 KB)
