# Supplementary figures and images for: Quest for Orthologs in the era of Data Deluge and AI: Challenges and Innovations in Orthology Prediction and Data Integration
Source: J Mol Evol. 2025 Oct 14;93(6):702–19. doi: 10.1007/s00239-025-10272-6 (PMC12756340; doi:10.1007/s00239-025-10272-6)

**(a)**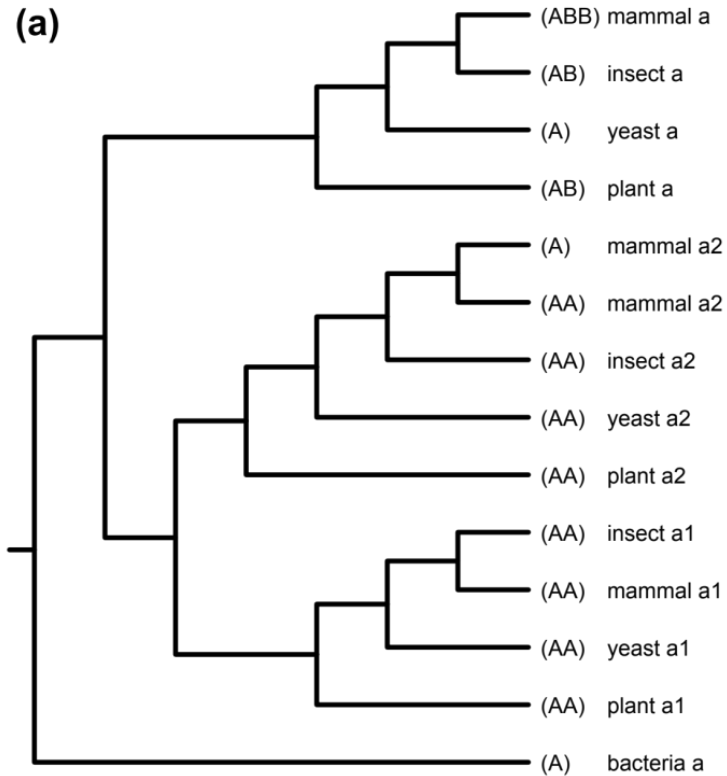**(b)**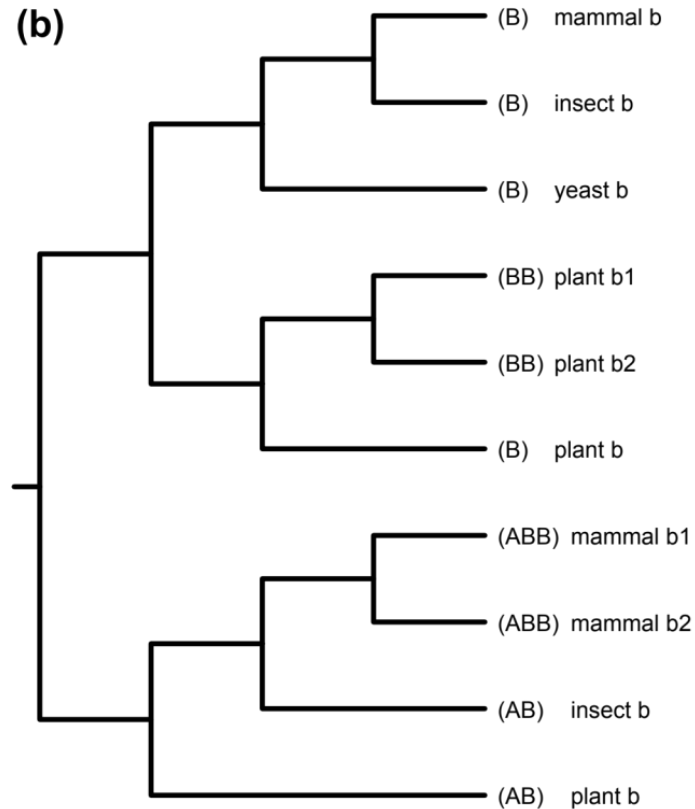

Supplement: Supplementary file 1 — Supplementary file1 (PDF 47 KB) [file 239_2025_10272_MOESM1_ESM.pdf]
